# Supplementary material for: HIV-1 Infection Results in Sphingosine-1-Phosphate Receptor 1 Dysregulation in the Human Thymus
Source: Int J Mol Sci. 2023 Sep 8;24(18):13865. doi: 10.3390/ijms241813865 (PMC10531245; doi:10.3390/ijms241813865)
Supplement: Supplementary file 1 [file ijms-24-13865-s001.zip › ijms-2557029-supplementary.pdf]

Supplementary Figures

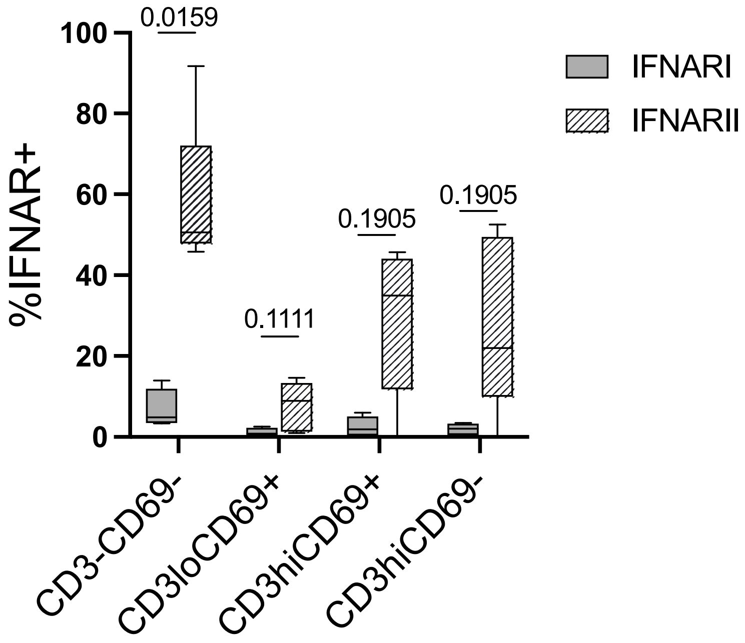

Supplementary Figure S1. IFNAR1 and IFNAR2 expression on human thymocyte subsets. Thymocyte subsets express higher levels of IFNAR2 (striped) than IFNAR1 (solid) *ex vivo*. Postnatal thymocytes were surface stained with antibodies to CD3, CD69, CD4, CD8, CD45RA, and CD27. IFNAR1 and IFNAR2 (**n=4 and n=5**, respectively) expression on immature CD3-CD69- and CD3lowCD69+ and mature CD3hiCD69+ and CD3hiCD69- is shown. Shown are the mean with the standard error of the mean (SEM). All comparisons were done by unpaired t-test.

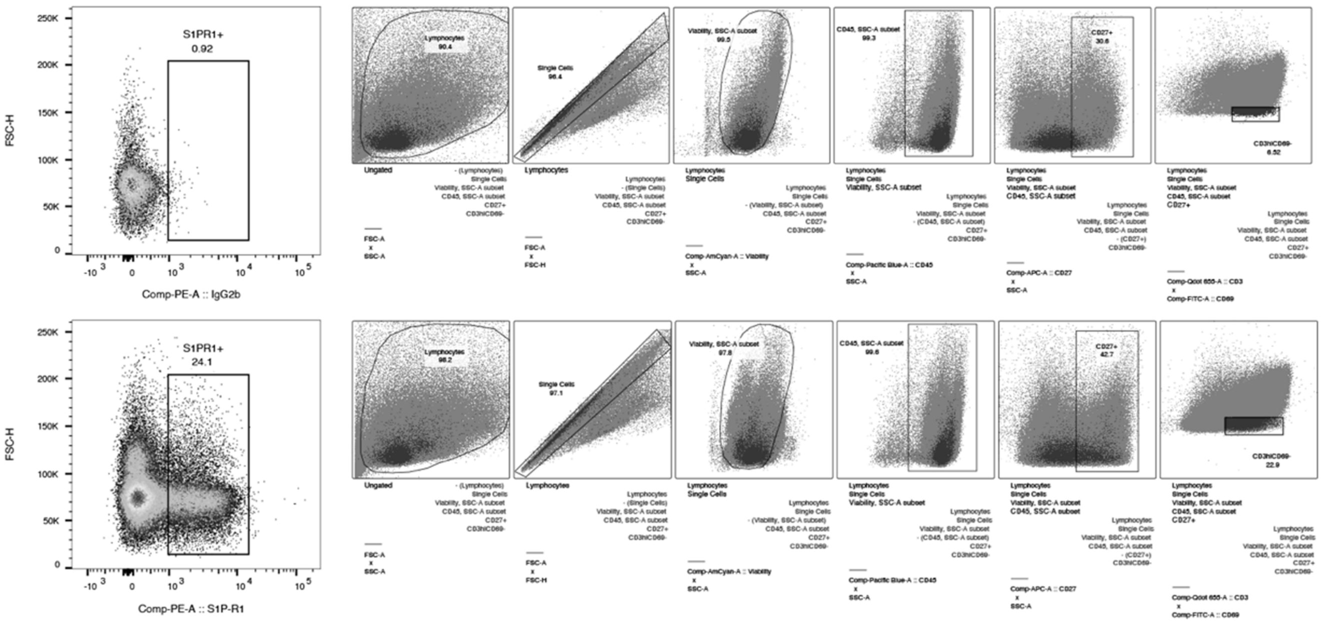

Supplementary Figure S2. Flow cytometry gating strategy for humanized mouse thymus analysis. The gating strategy used in mouse experiments examining the expression of S1PR1 is as follows: lymphocytes > single cells > live cells > CD45+ human cells > CD27+/- > CD3/69 populations > IgG2b/S1PR1.

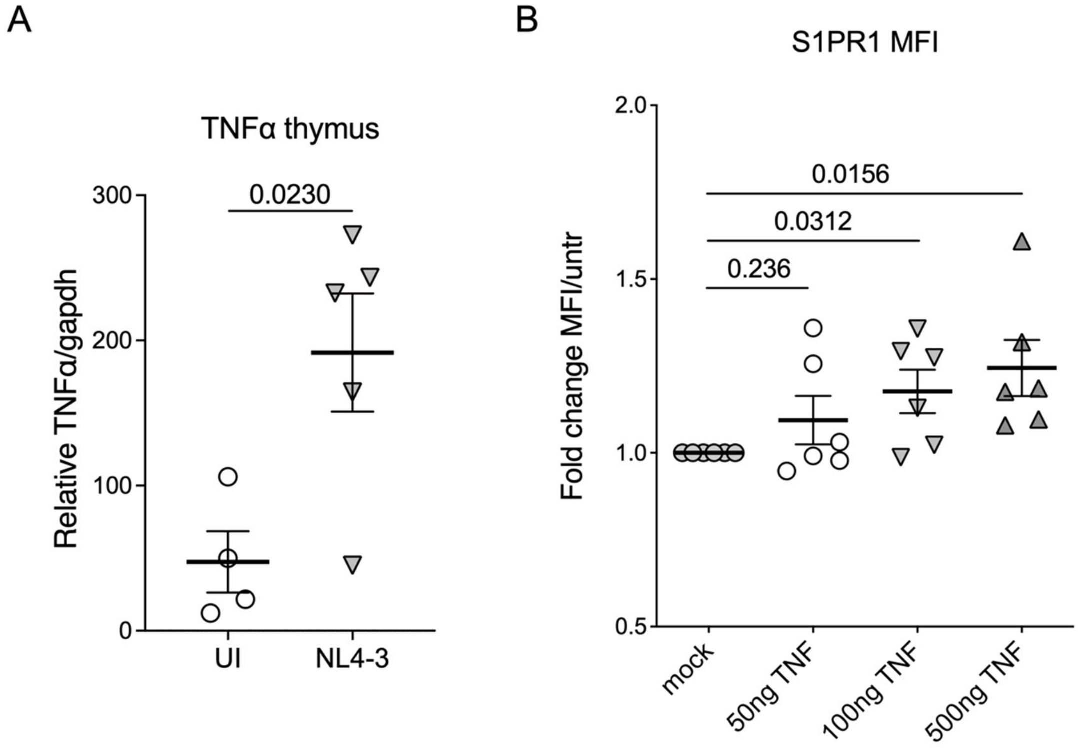

Supplementary Figure S3. Tumor necrosis factor alpha may contribute to S1PR1 upregulation following HIV infection. (**A**) Compared to mock (**n=4**) infected thy/liv implants, TNF-α mRNA is increased in the thy/liv implant 5-9 weeks post-infection with NL4-3 (**n=5**). Shown are relative copies of TNF-α mRNA per copies of the housekeeping gene GAPDH analyzed *ex vivo* at 9 weeks post-infection. (**B**) Exogenous TNF-α increases S1PR1 protein expression as measured by mean fluorescence intensity (MFI) on thymocytes *in vitro* to a statistically significant extent after TNF-α treatment at 100 and 500ng (**n=6**). Shown are the mean with the standard error of the mean (SEM). All comparisons were done by unpaired t-test.

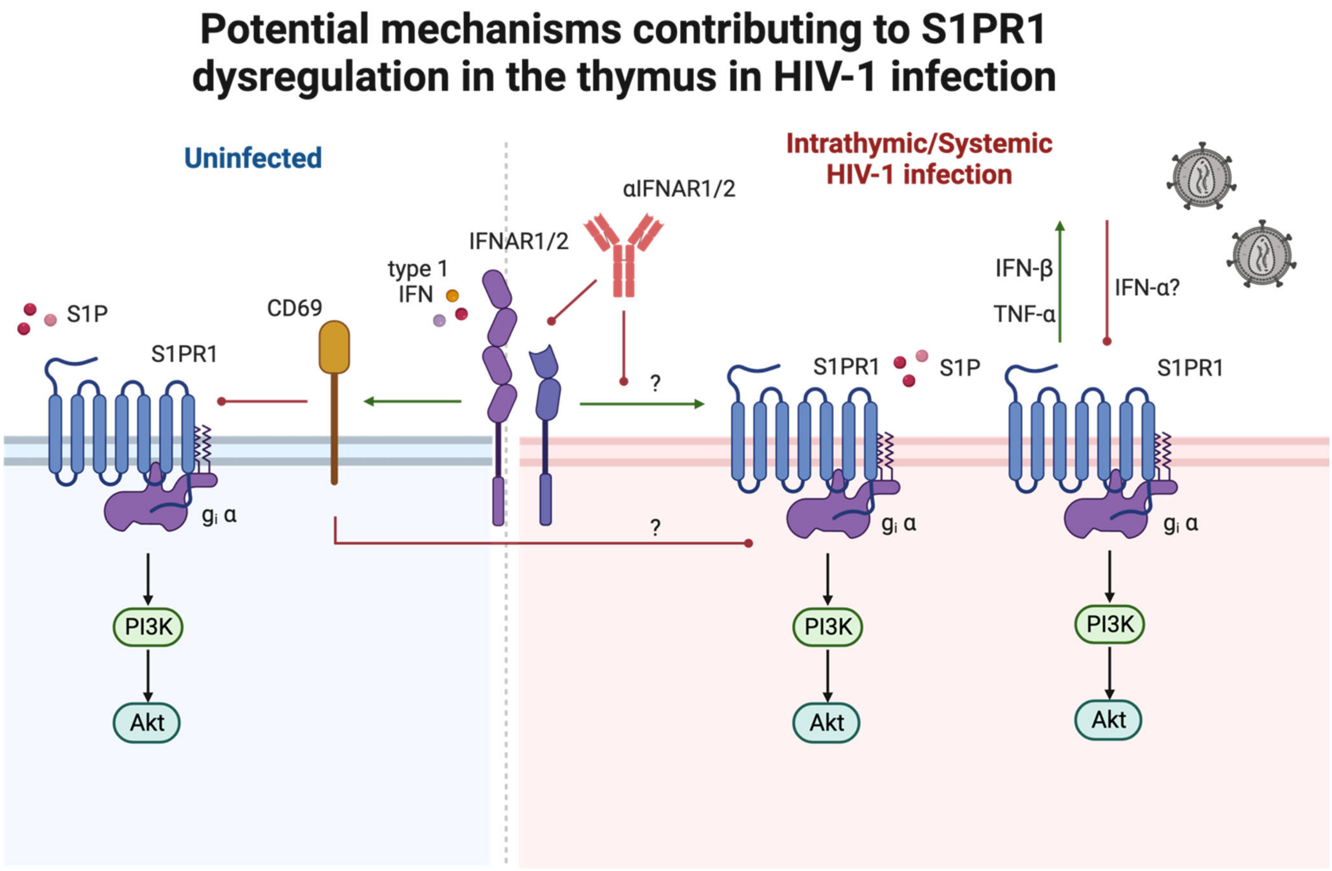

Supplementary Figure S4. Summary of potential mechanisms of S1PR1 dysregulation in thymocytes in HIV-1 infection. *The graphic was created on Biorender.com.*
